# In vitro antineoplastic effects of MK0752 in HPV-positive head and neck squamous cell carcinoma

**DOI:** 10.1007/s00432-023-05269-x

**Published:** 2023-08-16

**Authors:** Sara Varatanovic, Stefan Stoiber, Markus Haas, Alexander Lein, Lorenz Kadletz-Wanke, Faris F. Brkic

**Affiliations:** 1 Department of Otorhinolaryngology and Head and Neck Surgery, Medical University of Vienna, Vienna, Austria; 2 Department of Pathology, Medical University of Vienna, Vienna, Austria; 3 Christian Doppler Laboratory for Applied Metabolomics, Vienna, Austria

**Keywords:** MK0752, HPV, HNSCC

## Abstract

**Purpose:**

Gamma-secretase inhibitor MK0752 has shown a high therapeutic potential in different solid malignant tumors. Up to now, its antineoplastic effects were not investigated in head and neck squamous cell carcinoma (HNSCC) and particularly in human-papillomavirus (HPV)-positive tumors.

**Methods:**

We conducted cytotoxic, migration, and clonogenic assays in two HPV-negative HNSCC cell lines (Cal27 and FaDu) and one HPV-positive cell line (SCC154). Furthermore, in order to assess the pro-apoptotic effects of MK0752, a Caspase 3/7 Glo assay was performed.

**Results:**

Our experiments revealed antineoplastic effects of MK0752 in all three cell lines. Strong cytotoxic and antimigratory potential was shown in all cell lines, with strongest effects observed in the HPV-positive cell line. Meanwhile, anticlonogenic effects were only shown in Cal27 and SCC154. Most importantly, MK0752 induced apoptosis solely in HPV-positive SCC154.

**Conclusions:**

Our novel findings indicate a therapeutic potential of MK0752 in HPV-positive HNSCC. Indeed, further investigation is needed for validation of our results and for the assessment of the mechanistic background.

## Introduction

Head and neck squamous cell carcinoma (HNSCC) is the sixth most common malignancy. These tumors arise from pre-cancerous mucosa lesions in the oral, nasal, and paranasal cavities, pharynx, and larynx (Johnson et al. 2020). Importantly, despite globally declining incidences of HNSCC, a constant rise in the number of human-papillomavirus (HPV)-associated HNSCC cases has been observed in the last decades (WHO 2023). Besides being the prime risk factor for cervical cancers, it is also the trigger for approximately 25% of all HNSCC cases (Kobayashi et al. 2018). Interestingly, HPV-positive HNSCC has been shown to have better survival outcomes, mainly due to a better response to radiotherapy (Kobayashi et al. 2018). Still, a relevant subgroup of these patients have a dire prognosis (Brkic et al. 2021, 2022; Maier et al. 2023). A significant incidence rise of HPV-associated HNSCC cases is expected in upcoming years, potentially making the treatment of the specific high-risk group of HPV-positive HNSCC patients more vital (Brkic et al. 2022; Kobayashi et al. 2018; Maier et al. 2023). Therefore, new effective therapeutic options are urgently needed.

Gamma-secretase inhibitors (GSIs) are a class of small molecule inhibitors that prevent the cleavage of gamma-secretase substrates. MK0752 (MK) is a non-catalytic subunit of the gamma-secretase complex and catalyzes the intramembrane cleavage of integral membrane proteins such as the family of the Notch receptors and the amyloid-beta precursor protein (Uniprot 2023). For example, GSIs block the Notch signaling pathway by inhibiting the cleavage of Notch proteins which blocks their translocation to the nucleus. MK has been researched due to its antineoplastic effects observed in the treatment of different advanced solid cancers. In particular, it has shown promising results in pancreatic ductal adeno-carcinoma (Cook et al. 2018), breast cancer (Clinical trials 2019), T cell acute lymphoblastic leukemia/lymphoma (Deangelo et al. 2006), uterine leiomyosarcoma (Abedin et al. 2022), and other forms of advanced solid tumors (Ran et al. 2017). With regard to HNSCC, MK has been investigated in an HNSCC clinical trial in combination with ridaforolimus, with initially promising results. However, due to low tolerability of the treatment, the study has been discontinued (Piha-Paul et al. 2015). To the best of our knowledge, the antitumor potential of MK has not yet been assessed in HPV-positive HNSCC.

The Notch signaling pathway is a binary cell fate determinant, with its hyperactivation leading to the loss of its tumor-suppressive actions (Agrawal et al. 2011; Sun et al. 2014). Furthermore, Notch-1 mutations have been reported in 10–15% cases of HNSCC, making it the second most frequently mutated gene in this cancer entity (Fukusumi et al. 2018). It is suspected that Notch-1 mutations have a dual role for carcinogenesis. In particular, concurrent tumor suppressive and oncogenic properties have been reported (Agrawal et al. 2011).

HPV oncogenes are believed to disrupt Notch-mediated transcription of genes related to differentiation, which may be a contributing factor to carcinogenesis (Scarth et al. 2021). Furthermore, the HPV oncoprotein E6 can interact with the Notch signaling pathway resulting in the expression loss of Notch target genes, such as HEY and HES (Das et al. 2020). Therefore, a therapeutical potential of MK could be presumed for HPV-related HNSCC. Based on this, our aim was to investigate the antineoplastic effects of MK in HPV-positive HNSCC in vitro.

## Materials and methods

### Cell culturing and seeding

A total of three cell lines were used for all experiments, one HPV-positive cell line (SCC154) and two HPV-negative cell lines (FaDu and Cal27) (American Type Culture Collection, ATCC, Manassas, VA, USA). Cells were split at 80% confluence. In order to avoid de-novo mutagenesis, cells were not used after 30 passages. Protocol for cell culturing included Dulbecco’s modified eagle’s medium (DMEM), Penicillin/Streptomycin (P/S) and fetal calf serum (FBS) (Gibco, Thermo Fisher Scientific, Waltham, MA, USA). Cells were split by washing with Dulbecco’s phosphate-buffered saline (DPBS) (Gibco, Thermo Fisher Scientific, Waltham, MA, USA), followed by treatment with 0.05% trypsin–0.53 mM EDTA solution (Sigma-Aldrich, St. Louis, Missouri, USA). Cells were incubated in an environment of 37 °C and 5% CO_2_ (Hera Cell 240, Heraeus Holding GmbH) and cultivated in DMEM supplemented with 10% FBS and 1% P/S (“cell culture medium”). MK was acquired from Selleckchem (Houston, Texas, USA).

### Cytotoxicity assay

The cytotoxic effects of MK were evaluated in a dose–response assay. Cells were seeded into 96-well plates (Sarstedt, Nurnbrecht, Germany). SCC154 (10,000 cells/well), FaDu and Cal27 (5000 cells/well) were seeded in 100 μL DMEM and incubated for 24 h. Following the incubation, cells were exposed to increasing concentrations of MK, previously dissolved in dimethyl sulfoxide (DMSO) (Sigma-Aldrich, St. Louis, Missouri, USA). The inhibitor was diluted in cell culture medium in following concentration ranges: 3.75–60 μM for FaDu and Cal27 and 2.5–40 μM for SCC154 cells. For each dose and the vehicle control (0.1% DMSO), five replicates were obtained. Besides MK treatment, cells were irradiated at 2, 4, and 8 Gray (Gy) utilizing the YXLON device (YXLON, International GmbH, Hamburg, Germany). After treatment with MK and radiotherapy, cells were incubated for 72 h. Next, the medium was removed and replaced with 100 μL of a 56 μM resazurin (Sigma-Aldrich, St. Louis, Missouri, USA) solution. FaDu and Cal27 cell lines were incubated for 90 min, while SCC154 were incubated for 3 h. Absorbance measurements were performed using the TECAN Spark reader (TECAN Spark, Tecan Group Ltd, Maennedorf, Switzerland). The half maximal inhibitory concentration (IC50) of MK was calculated and used for subsequent experiments.

### Migration assay

The migration assay was performed using 24-well plates (Greiner Bio-One, Frickenhausen, Germany) with ibidi inserts (Greiner Bio-One, Frickenhausen, Germany) for the initial gap creation. SCC154 (250,000 cells/ibidi chamber) and FaDu and Cal27 (70,000 cells/ibidi chamber) were seeded. Inserts were removed after cells reached 100% confluency. A starvation medium (DMEM with 1% FBS and 1% P/S) was used in order to limit the proliferation of the cells. Following the removal of the ibidi inserts, cells were washed and the starvation media containing MK at IC50 doses (33 μM for FaDu and Cal27, 25 μM for SCC154 cell line) and the vehicle control (0.1% DMSO) were added. Effects of MK treatment on migration were analyzed using microscoping imaging using the TECAN Spark plate reader at the following time points: 0, 24, and 48 h. Analysis of the images and calculation of the percentage of remaining gap area at each time point, compared to baseline, were performed using ImageJ (https://github.com/imagej/ImageJ).

### Colony formation assay

The anticlonogenic potential of MK was evaluated in a colony formation assay. In 12-well plates (Sarstedt, Nurnbrecht, Germany), 250 FaDu cells/well and 250 Cal27 cells/well 1000 SCC154 cells/well were seeded in 1 mL of cell culture medium. Following 24-h incubation, cells were treated with MK at the respective IC50 concentrations and incubated for 72 h before exchanging the media. Then, SCC154 and Cal27 were incubated for additional 10 days and FaDu for 14 days. Finally, cells were washed with DPBS and colonies were imaged using the TECAN Spark plate reader. Colonies were counted using ImageJ.

### Cell apoptosis assay

To evaluate the pro-apoptotic effect of MK, a Caspase 3/7 Glo Kit (Promega, Fitchburg, Wisconsin, USA) was used. The Caspase 3/7 Glo reagent was prepared according to the manufacturer’s instructions. Next, 96-well plates (Sarstedt, Nurnbrecht, Germany) were seeded with 7500 FaDu and Cal27 cells/well and 15,000 SCC154 cells/well in 100 μL DMEM. After 24 h of incubation, the cells were treated with the respective IC50 of MK and a vehicle control. After 72 h, the medium was collected and mixed in 1:1 ratio with the Caspase 3/7 Glo reagent in three following conditions: blank reaction (Caspase Glo 3/7 reagent, vehicle and cell culture medium without cells), negative control (Caspase Glo 3/7 Reagent and vehicle-treated cells in medium) and assay (Caspase 3/7 Glo Reagent and MK-treated cells in medium). After 30 min of incubation at room temperature, luminescence was measured using TECAN Spark reader.

### Statistical analysis

The results were normalized to the vehicle control in all assays. The cytotoxic effects were compared with a two-way ANOVA. Furthermore, results obtained in the migration and colony formation were analyzed with a one-way ANOVA. Apoptosis assay results were analyzed with the *t*-test. GraphPad Prism version 8.0.0 for Windows (GraphPad Software, San Diego, California USA, www.graphpad.com) was used for the statistical analysis and graphical representation. Figures show mean values ± standard deviation (SD). Synergy finder (synergyfinder.com) was used to evaluate synergistical effect of the GSI MK and the irradiation treatment utilizing a zero-interaction potency (ZIP) model. ZIP scores below 10 were considered antagonistic, between − 10 and 10 additive, and above 10 as synergistic.

## Results

### MK decreases cell viability in all three HNSCC cell lines

**Fig. 1:**
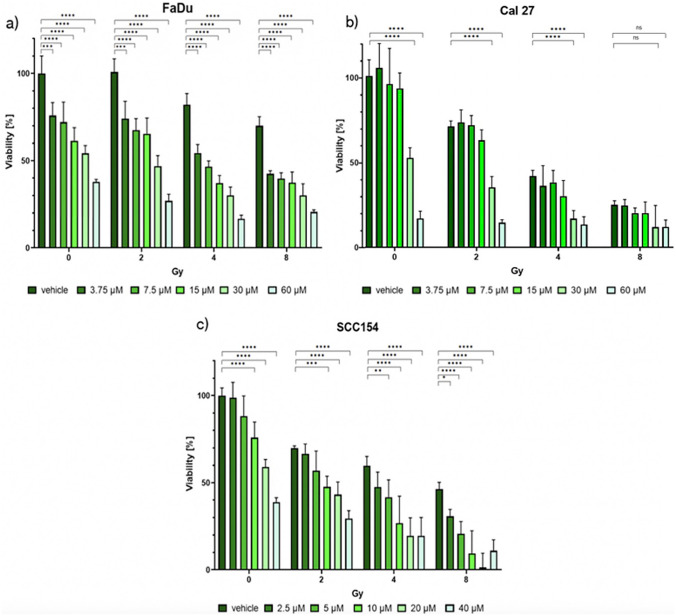
Cytotoxicity assay showing cell viability of FaDu (**a**), Cal27 (**b**), and SCC154 (**c**) cell lines after treatment with MK and concurrent radiation therapy. Highest antineoplastic potential was shown in SCC154. Meanwhile, less potent antiproliferation effects were observed in two HPV-negative cell lines. *Gy* Gray. Significant differences between controls and MK0752-treated cells are represented with asterisks (*p < 0.05, **p < 0.01, ***p < 0.001, ****p < 0.0001)

We observed significant antiproliferative effects of MK in HPV-negative and HPV-positive HNSCC cell lines (Fig. 1). Interestingly, MK showed the highest cytotoxic potential in the HPV-positive HNSCC cell line (Fig. 1c). When treating FaDu and Cal27 with 60 μM of MK and SCC154 with 40 μM, 20%, 10%, and 10% of viable cells were observed, respectively. In particular, calculated IC50 values were 33 μM for Cal27 and FaDu and 25 μM for SCC154 cells.

**Fig. 2:**
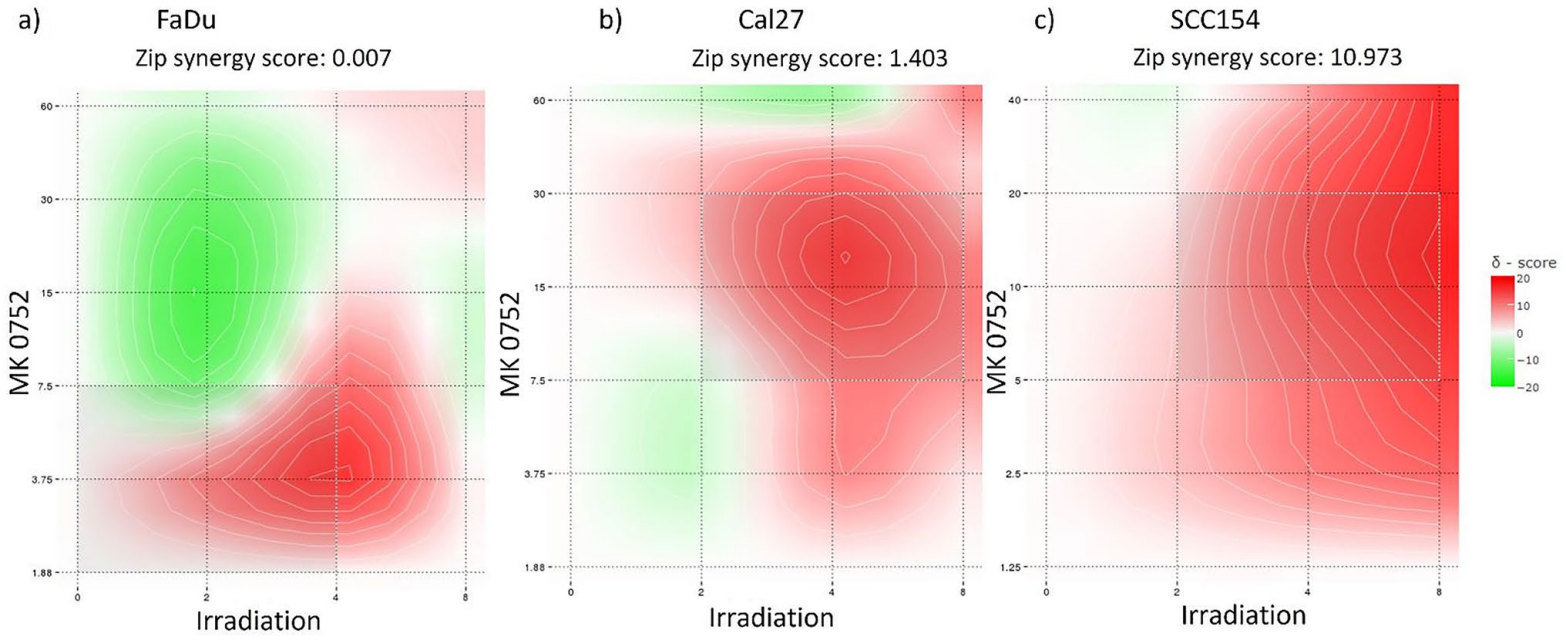
Synergy scores of irradiation treatment and MK treatment. Maps of ZIP scores for Cal27 (**a**), FaDu (**b**), and SCC154 cell line (**c**). Synergy is defined as excess response due to drug and radiation interactions, whereby scores higher than 10 can be interpreted as synergistic interactions, scores between 10 and − 10 suggest additive effects, and scores under − 10 indicate antagonistic effects (Chen et al. 2016). While synergistic and additive effects were shown in all three cell lines, particularly high rates of synergistic effects of MK with radiotherapy were observed in SCC154 and Cal27

Furthermore, synergistic effects between MK treatment and radiotherapy were assessed (Fig. 2). Additive and synergistic effects were shown in all cell lines (Fig. 2a–c), and particularly, strong synergistic effects were observed in Cal27 and SCC154.

### MK demonstrates antimigratory potential in HNSCC cell lines

**Fig. 3:**
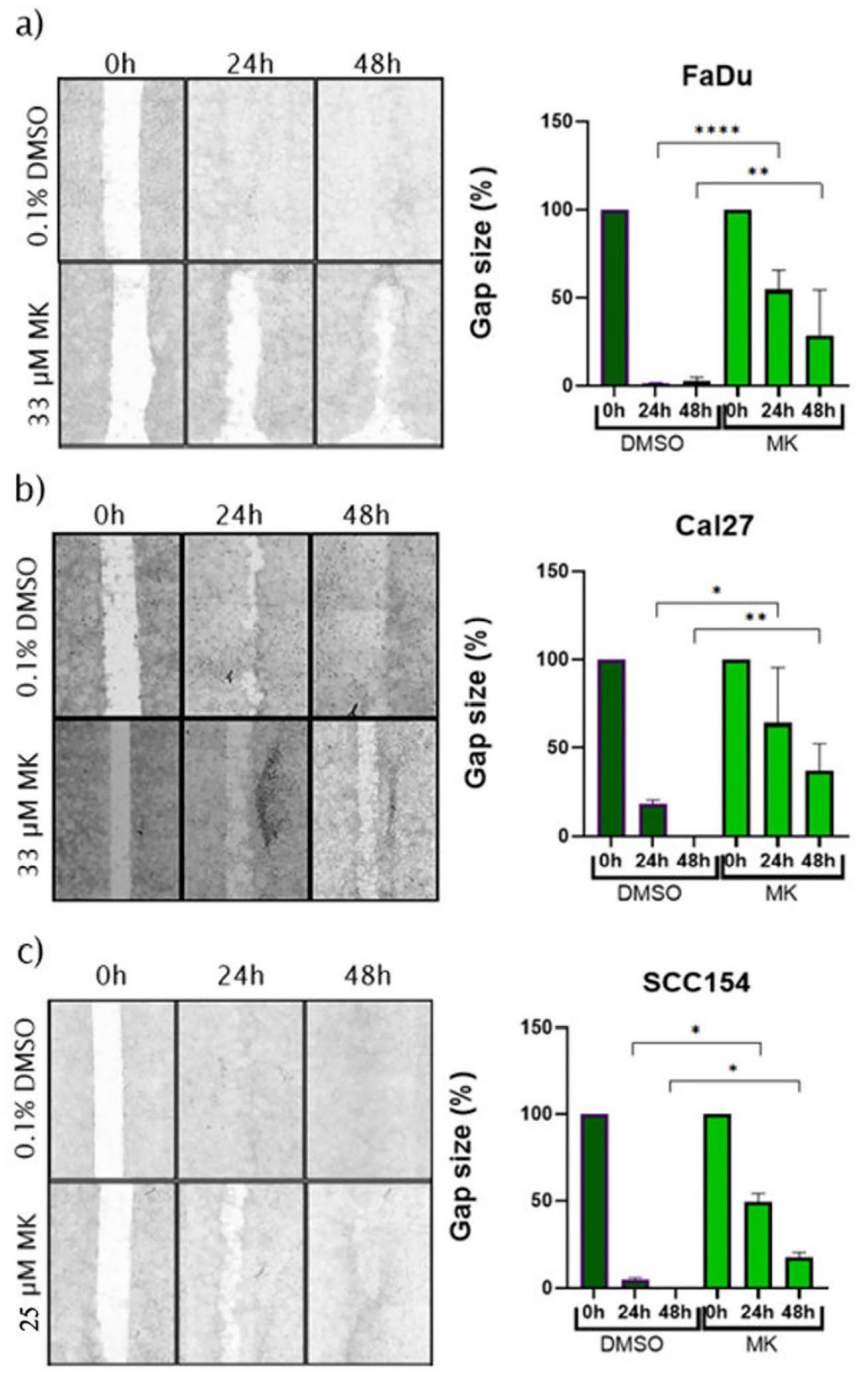
Migration assay showing the antimigratory potential of MK in FaDu (**a**), Cal27 (**b**), and SCC154 (**c**) cell lines. While strong antimigratory effects were shown in all three cell lines, MK was particularly potent in HPV-negative cell lines. Significant differences between controls and MK-treated cells are represented with asterisks (*p < 0.05, **p < 0.01, ***p < 0.001, ****p < 0.0001)

The previously determined IC50 of MK was used in the migration assay. As shown in Fig. 3, MK shows antimigratory potential in all three cell lines, with lower MK concentration in SCC154. While control group in in all three cell lines showed over 90% gap closure at 24-h time point, the gaps remained open in treated cells at 24-h and 48-h time-points. In particular, MK had stronger antimigratory effect on FaDu and Cal27 cell line and there was still significant difference in SCC154 gap closures as well.

### MK exerts anticlonogenic effects in HNSCC cell lines

**Fig. 4:**
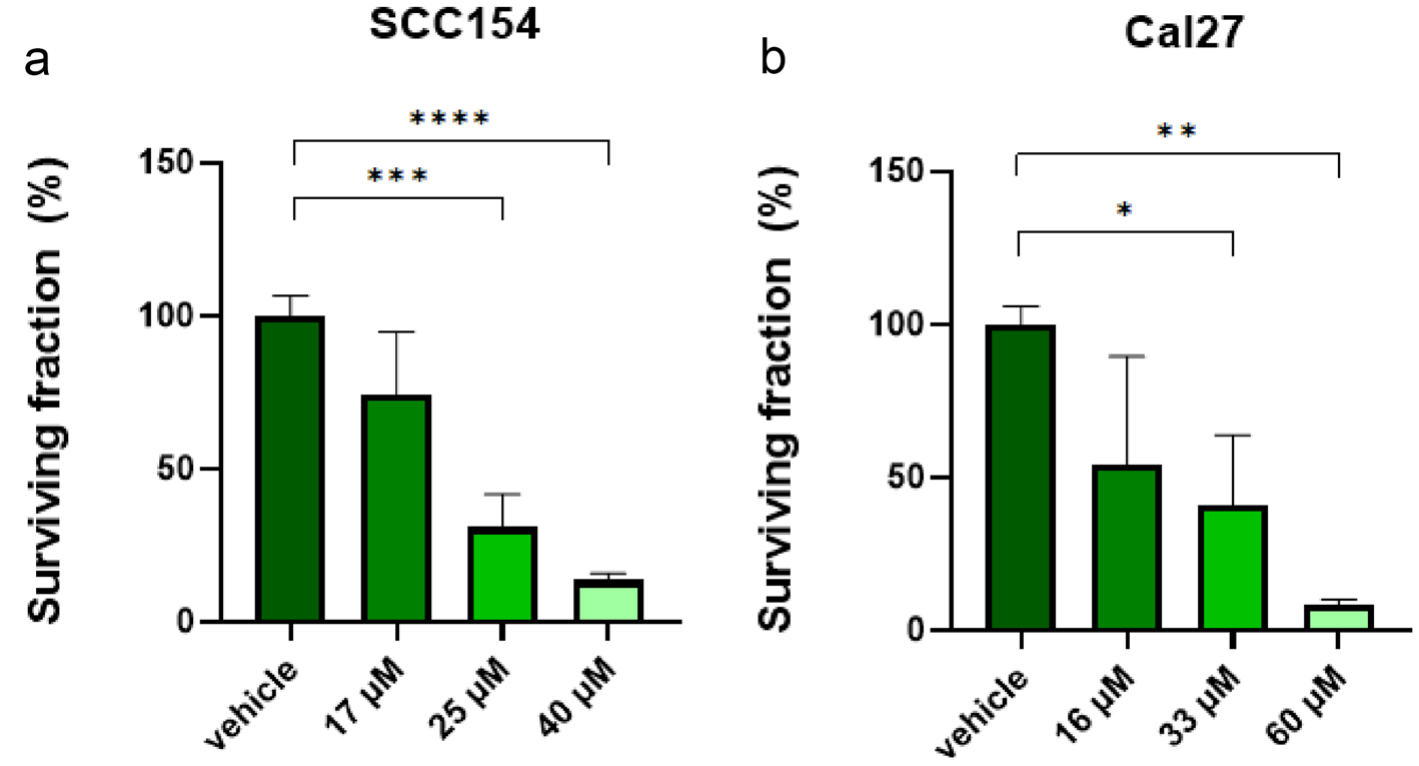
Colony-forming assay showing the anticlonogenic effects of MK in SCC154 (**a**) and Cal27 (**b**) cell lines. Similar anticlonogenic potential was observed in HPV-negative and -positive HNSCC cell lines. Statistical results are depicted with asterisks (*p < 0.05, ***p < 0.001, ****p < 0.0001)

The colony formation assay indicated that the colony formation capability was suppressed by MK in SCC154 and Cal27 cell lines (Fig. 4). Meanwhile, for the FaDu cell line no reproducible results could be generated and was therefore excluded from the analysis. Importantly, the anticlonogenic effect of the IC50 concentration of MK were potent in SCC154, where the colony quantity lowers gradually with increasing concentrations. Similarly, colony quantities decreased with increasing inhibitor concentration in Cal27. Treatment of SCC154 with 40 μM and Cal27 with 60 μM resulted in surviving fractions of less than 10% of cells.

### MK induces apoptosis solely in the HPV-positive HNSCC cell line

**Fig. 5:**
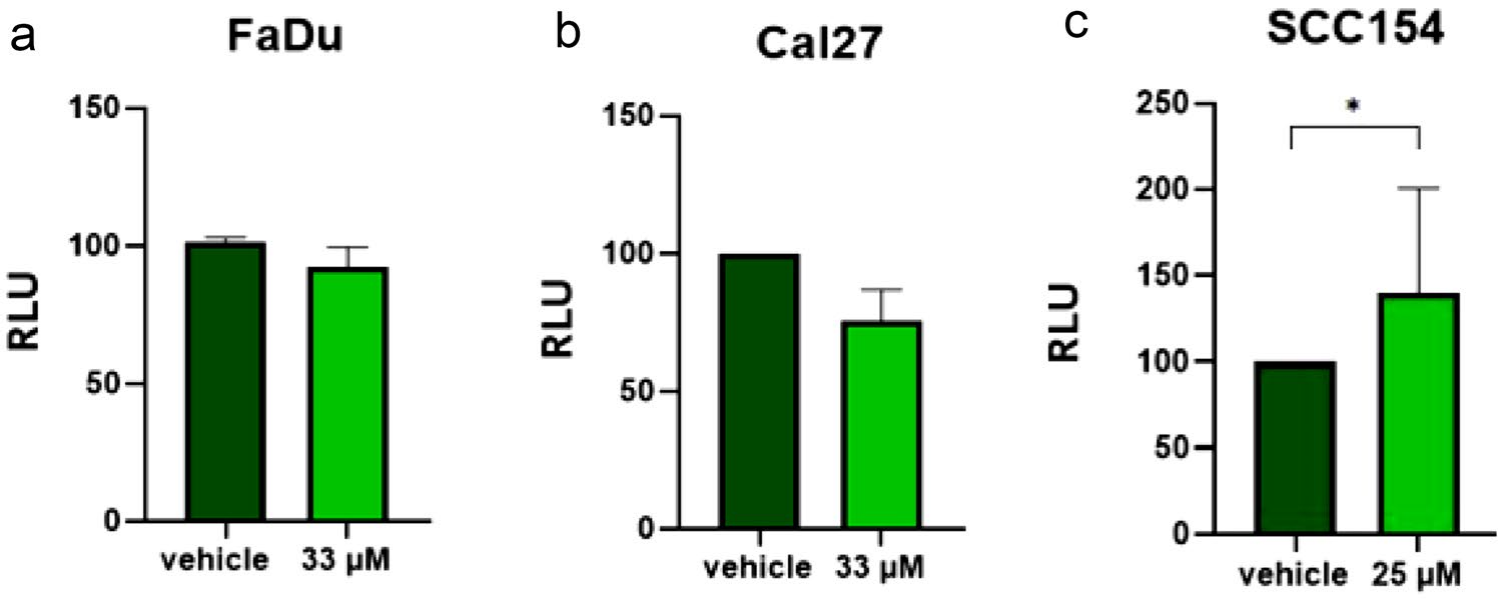
Apoptosis assay in FaDu (**a**), Cal27 (**b**), and SCC154 (**c**) cells after treatment with MK and vehicle control. Proapoptotic effects were observed only in SCC154 (**c**), while no apoptosis was induced in two HPV-negative cell lines. Statistical results are depicted with asterisks (*p < 0.05)

Lastly, we assessed the pro-apoptotic effects MK on all three cell lines. Cells were treated with MK at the corresponding IC50 for 72 h, and the cell apoptosis was assessed with the Caspase 3/7 Glo assay. As shown in Fig. 5, apoptosis was only induced in SCC154 (Fig. 5c). The mean quantity of relative light units (RLU) has decreased in FaDu and Cal27 cell lines to ≈90% and ≈80%, respectively. In comparison, SCC154 cell line has had an increase to ≈140%, on average (Fig. 5a–c).

## Discussion

The current study provides first insights into the antitumor properties of MK on HNSCC cells in vitro. In particular, MK has shown cytotoxic, antimigratory, and anticlonogenic potential in HNSCC. Interestingly, pro-apoptotic effects were only observed in the HPV-positive cells. Generally, the observed antineoplastic effects were more potent in the HPV-related cell line.

The Notch signaling pathway regulates many aspects of cancer biology, such as stem cell renewal, proliferation, tumor angiogenesis, and metastasis. Its association with tumorigenesis of different solid tumors is well-established. Interestingly, the findings with regards to involvement of the Notch pathway and the therapeutic potential in HNSCC are polarizing (Sun et al. 2014). Zeng et al. (2005) showed that HNSCC cells with overexpressed Jagged 1 (ligand of the pathway) have enhanced neovascularization and tumor growth. Furthermore, Lin et al. (2010) showed an association of high-level co-expression of Jagged-1 and Notch-1 with poor survival in patients with HNSCC. They observed that interaction between Jagged-1 and Notch-1 constitutively activates Notch signaling, resulting in a more aggressive phenotype. On the contrary, another group showed that the loss of Notch signaling, as well as the presence of HPV oncogenes E6 and E7, is associated with increased likelihood of HPV-positive tumor incidence and grade (Nyman et al. 2018).

Interestingly, Fukusumi and Califano (2018) described different effects of Notch proteins on HNSCC. They concluded that Notch-1 plays a bimodal role in HNSCC. Notch-1 was shown to have tumor-suppressive and oncogenic properties depending on inactivating or activating mutations. They detected higher Notch-2 expression in HNSCC cases in comparison to healthy tissue, as well as a 4% mutation rate for the Notch-3 gene and a slightly increased expression of Notch-4. In summary, the role of Notch signaling in HNSCC seems to be bimodal, on the one hand tumor suppressive with inactivating mutations and on the other hand oncogenic with activating and upregulating mutations, and requires further elucidation.

In the current study, we observed cytotoxic effects of MK in all tested HNSCC cell lines. Similar observations in ovarian cancer were reported by Chen et al. (2016). Furthermore, a MK-induced decrease in cell viability was also observed in a study by Abedin et al. (2022). Additionally, breast cancer tumor growth was shown to be suppressed by MK (Schott et al. 2013). Up to date, no studies were reported on synergy of MK and radiotherapy. Therefore, our results showing additive and synergistic effects of the inhibitor and concurrent irradiation are novel and particularly relevant due to well-known high sensitivity of HPV-positive HNSCC to radiation therapy (Brkic et al. 2022; Kobayashi et al. 2018).

Furthermore, we observed antimigratory effects of MK. Similar observations were reported in a study on colon cancer stem cells (Yuwei et al. 2022). Moreover, the antimigratory potential of MK has also been noted in a study from Saltarella et al. (2019) on multiple myeloma cells showing significantly reduced spontaneous and chemotactic migration after treatment with MK.

The results in our study imply that the apoptosis is induced only in the HPV-positive HNSCC cell line. Meanwhile, observed antineoplastic effects in FaDu and Cal27 were seemingly not directly dependent on caspase 3/7-mediated apoptosis. Therefore, an interaction between HPV infection and gamma-secretase or the Notch pathway in HNSCC can be presumed. As previously mentioned, no other studies analyzed the effects of MK in HPV positive tumors yet. However, similar pro-apoptotic effects of MK were reported in T cell leukemia (Greene et al. 2016) and pancreatic cancer cells (Abel et al. 2014).

Several authors reported the possible interactions of HPV infection and Notch signaling. A study by Rampias et al. (2014) indicated that HPV has repressive effects on the Notch pathway. Furthermore, a study by Vliet-Gregg et al. (2015) noted that high-risk HPV oncogenes lead to a higher expression of Notch-1 and increased canonical pathway activation. Furthermore, Lim et al. (2022) indicated the relevance of different HPV strands with regard to the Notch pathway. Their research showed repression of the Notch pathway by beta HPV E6 proteins, while alpha HPV E6 proteins activated the pathway. However, as the data on the interaction of HPV in HNSCC with the Notch pathway remain sparse, further investigations are warranted to understand the mechanistic background behind our findings, particularly the fact that pro-apoptotic effects were solely observed in the HPV-associated cell line.

Our novel findings are limited by several shortcomings. First, all experiments were con-ducted only in 2D cell culture experiments and on established HNSCC cell lines. Therefore, effects of the 3D tumor architecture and the tumor microenvironment are missing. Furthermore, the mechanistic background behind our findings remains unclear and needs further elucidations. Lastly, only one HPV-associated cell line was used.

In conclusion, our study provides novel results and imply that MK might have a therapeutic potential in HPV positive HNSCC. However, further experiments are needed, particularly in additional HPV-positive cell lines, in order to validate our findings.

## Data Availability

The public dataset supporting the conclusions of this article is available from the corresponding author upon reasonable request.

## Abbreviations

HNSCC: Head and neck squamous cell carcinoma
HPV: Human papillomavirus
MK: MK0752
DMEM: Modified eagle’s medium
P/S: Penicillin/streptomycin
FBS: Fetal calf serum
DMSO: Dimethyl sulfoxide
IC50: Half-maximal inhibitory concentration
